# Low-Tension Glaucoma: An Oxymoron in Ophthalmology

**DOI:** 10.5888/pcd16.180534

**Published:** 2019-01-24

**Authors:** Ciro Costagliola, Luca Agnifili, Leonardo Mastropasqua, Alfonso di Costanzo

**Affiliations:** 1 Department of Medicine and Health Sciences, University of Molise, Campobasso, Italy; 2 Department of Medicine and Aging Science, Ophthalmology Clinic, University G. d’Annunzio of Chieti-Pescara, Chieti, Italy

The term glaucoma refers to a group of ocular conditions characterized by progressive optic nerve damage and loss of visual field (1). Glaucomatous optic neuropathy is due to the progressive loss of retinal ganglion cells; elevated intraocular pressure (IOP) is one major risk factor. IOP may act directly, by a mechanical effect, or indirectly, by influencing blood supply (2). Factors that influence the progression of glaucomatous optic neuropathy include older age, advanced stage of disease, higher IOP, and disc hemorrhages (3). In patients with primary open-angle glaucoma (POAG), the increased resistance to aqueous outflow through the trabecular meshwork is the major culprit for elevated IOP. However, despite adequate control of IOP, glaucomatous optic neuropathy may frequently continue to progress. Thus, factors not related to IOP are recognized, with the most important being a decrease in blood supply to the optic nerve (4).

Other factors not related to IOP include glutamate toxicity, oxidative stress, autoimmunity, and vascular dysregulation (3). Low-tension glaucoma (LTG) is defined as a form of glaucoma that closely mimics POAG, but IOP levels are within the normal range and the probable pathogenesis is vascular.

The controversial questions are 1) is LTG a disease on the spectrum of POAG (on the left side of the distribution of IOP, at the lowest levels)?, 2) is the optic disc appearance secondary to optic nerve hypoperfusion due to vascular diseases?, or 3) should LTG be included in a spectrum of congenital and acquired optic neuropathies that can simulate glaucomatous optic neuropathy?

## Is Low-Tension Glaucoma a Disease on the Spectrum of Primary Open-Angle Glaucoma?

When in 1857 Albrecht von Graefe described a form of glaucoma manifesting damage to the optic nerve head and an open anterior chamber angle, with IOP within the reference range, digital palpation tonometry was considered the gold standard. If he had used an impression tonometer to measure IOP, the major shortcoming of the tonometry would have been that it displaced so much fluid upon contact with the eye that the measured readings would be highly variable and inaccurate. Conversely, if he had used indentation tonometry, it would not have considered the misclassification resulting from the presence of a thin central corneal (1). Many diagnoses of LTG were for eyes with thin corneas and were based on false low values for IOP, a finding that casts some doubts on the diagnosis of LTG (5). Furthermore, the absence of elevated IOP must be found on measurements performed more than one time or during daytime, since IOP shows diurnal and nocturnal fluctuations in healthy subjects, and even more in patients with POAG or LTG (6). For variations in IOP, 3 populations of LTG patients may be distinguished: patients without IOP fluctuations, patients with diurnal IOP acrophase (the crest or peak of a cycle), and patients with nocturnal IOP acrophase (7). Patients in the 2 last categories should be considered true POAG patients rather than LTG patients, in whom glaucomatous optic neuropathy occurs despite normal IOP. It is likely that patients with a diurnal or nocturnal acrophase have been enrolled in studies based on the mechanical theory; reduction of IOP might slow down the progression of visual field loss only in these patients (8). Data from the Low-Pressure Glaucoma Treatment Study highlighted the role of IOP in LTG pathogenesis; the progression of visual field loss was reduced by 9.1% with timolol 0.5% and by 39.1% with brimonidine 0.2% after 2 years of treatment (8). However, in this study, IOP values were recorded exclusively during daytime. Thus, whether patients with a worse visual field had IOP nocturnal acrophase is unknown. Other studies on asymmetric LTG reported that the eye with higher IOP shows greater glaucomatous damage than the eye with lower IOP, which sustains the role of IOP in the pathogenesis (9). Yet, in all these clinical trials IOP was measured only during office hours; thus, the behavior of nocturnal IOP was not recorded. A recent study in which nychthemeral IOP curves were evaluated with a telemetric sensor showed a nocturnal acrophase with IOP spikes in patients with LTG, although these spikes were at significantly lower levels than the spikes found among patients with POAG (7). This study reported that IOP peaked at night in 40% to 80% of patients with normal-tension glaucoma, and the pattern in these patients was similar to the pattern in patients with POAG. In 24-hour curves, patients with LTG and POAG had more pronounced patterns of IOP in the evening and night than in the morning, with more peaks and greater IOP fluctuation during the night than during the evening (7). Other factors may corroborate the hypothesis of a primary IOP-related mechanical stress in LTG, such as the presence of changes in the aqueous humor outflow pathways similar to those occurring in POAG (10). 

In patients where LTG can be considered a disease on the spectrum of POAG, diagnostic and therapeutic strategies similar to those for patients with hyperbaric glaucoma must be implemented, with the help of an ophthalmologist (Figure).

**Figure:**
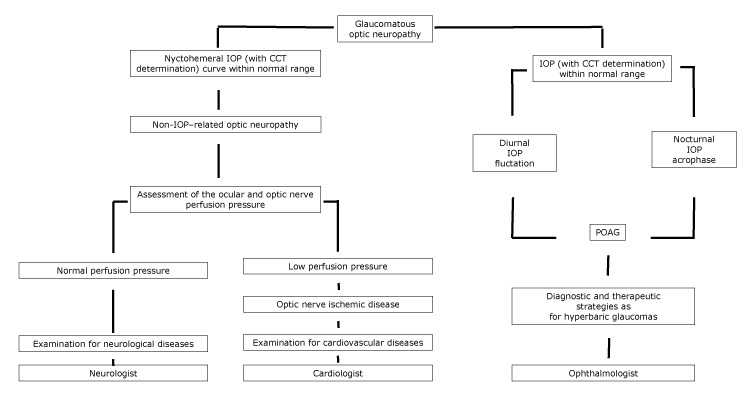
Proposed flowchart of optic neuropathy, with specialist referral, according to the nychthemeral IOP characteristics. Abbreviations: CCT, central corneal thickness; IOP, intraocular pressure; POAG, primary open-angle glaucoma.

## Is the Optic Disc Appearance Secondary to Optic Nerve Hypoperfusion Due to Vascular Diseases?

In patients with LTG and normal nychthemeral IOP curves, mechanical factors cannot be responsible for optic neuropathy; pressure-independent factors may be involved, with vascular alterations potentially being the most important (5). A glaucomatous-like optic neuropathy may be observed in patients with history of a cardiovascular event or with chronic atherosclerosis or obstructive arterial diseases (11). Reduced blood flow velocity in retrobulbar arteries and in cerebral circulation, low diastolic blood pressure, and smaller central retinal vessel diameter have been also observed in patients with LTG (5). In these patients, the pathogenesis of disease is due to an impaired ocular perfusion pressure, mainly linked to primary vascular dysregulation or to a generalized dysfunction of the endothelial or autonomic nervous system (5,11). However, it is unclear whether these factors were the cause or the result of the optic neuropathy. From a clinical point of view, LTG shows a higher incidence of disc hemorrhages and is more frequently associated with vascular diseases such as migraine, obstructive sleep apnea, or Raynaud’s syndrome than with IOP (5). Finally, about half of LTG patients with pre-perimetric disease show damage progression despite normal IOP values (12). All these findings suggest the important role of vascular dysregulation.

In patients with cardiovascular diseases, optic disc cupping may tend not to progress if the underlying cause of the optic neuropathy has been controlled. Moreover, LTG patients may show circumpapillary atrophy as well as cerebral cortical microinfarcts, which are signs of ischemia (5). In patients where vascular diseases induce a optic nerve hypoperfusion, a complete diagnostic examination for cardiovascular diseases must conducted, with the help of a cardiologist (Figure).

## Should LTG Be Included in a Spectrum of Congenital and Acquired Optic Neuropathies That Can Simulate a Glaucomatous Optic Neuropathy?

When the more frequent causes of optic disc cupping have been excluded, the possibility of a neurodegenerative optic neuropathy should be considered. These cases are the most frustrating, because optic disc damage progresses even after IOP has been lowered. Many congenital and acquired optic neuropathies are included in this group of cases, and differentiation between glaucomatous and nonglaucomatous cupping can be challenging even for experienced observers (13). Among the congenital forms of optic disc cupping, megalopapilla, autosomal dominant optic atrophy, and Leber hereditary optic neuropathy could produce an optic disc excavation simulating glaucomatous optic neuropathy.

The acquired neuropathies might be secondary to inflammatory, compressive, toxic, and traumatic causes. Optic neuritis may produce an increase of the cup-to-disc ratio that, although unilateral, may be confused with glaucomatous optic neuropathy. Compressive lesions including meningioma, pituitary adenoma, craniopharyngioma, and internal carotid artery aneurysm may lead to asymmetric optic disc cupping and erroneously attributed to LTG. Methanol and ethambutol poisoning might produce a bilateral optic disc cupping similar to glaucomatous optic neuropathy, secondary to axonal loss. Thus, when an asymmetric enlarged cup is observed, further neuro-ophthalmologic investigation is necessary (13).

Lastly, among conditions leading to optic disc cupping, aging has a main role. Harju et al found that the degree of optic disc cupping increased in healthy older study participants because of physiological fiber loss. Their study population was appropriate and represented a set of healthy eyes without glaucoma; throughout 11 years of follow-up, no study participants developed visual field changes, and no significant rise in IOP was recorded (14). In this latter case, a complete diagnostic examination for neurological diseases must be conducted, with the help of a neurologist (Figure).

Improvements in diagnostic techniques make it easier than before to classify optic disc cupping; a description of optic disc characteristics combined with the imaging of the retinal nerve fiber layer and optic disc topography permits differentiation between glaucomatous and nonglaucomatous optic disc cupping. Moreover, a careful analysis of patient history, together with morphologic and functional assessment of the optic nerve, helps to identify disorders.

The term LTG could be an oxymoron, a nostalgic memory of the past when a defined diagnosis was not possible. Using the term is like using the word “fever” when there is no high temperature or saying *festina lente* (“more haste, less speed”). The term LTG may be misleading or inaccurate, because it refers to a mechanical problem of IOP, whereas optic disc cupping and visual field loss in eyes with normal intraocular pressure are caused by other factors. Therefore, in the presence of optic disc cupping with normal IOP, ophthalmologists should investigate other plausible causes of optic nerve damage besides intraocular pressure.
